# OLDER ADULT RESEARCH SPECIALISTS SEEK ENCORE CAREERS PROMOTING THE INCLUSION OF OLDER ADULT PEERS IN CLINICAL TRIALS

**DOI:** 10.1093/geroni/igad104.0555

**Published:** 2023-12-21

**Authors:** Kathryn Nearing, Jodi Waterhouse

**Affiliations:** University of Colorado Anschutz Medical Campus, Denver, Colorado, United States; University of Colorado Anschutz Medical Campus Multi-disciplinary Center on Aging, Aurora, Colorado, United States

## Abstract

The University of Colorado Multi-disciplinary Center on Aging trains and promotes the hiring of older adults from diverse backgrounds to pursue encore careers as Older Adult Research Specialists (OARS). Health navigation training is the foundation of these research navigators’ unique preparation for paid positions that focus on recruiting and retaining older adult peers in clinical trials. Specifically, OARS’ roles include: 1) developing effective communication and outreach strategies to engage older adult peers in research; 2) recruiting and consenting participants for clinical trials; 3) supporting retention by identifying barriers to study participation and connecting research participants with needed community-based resources/support; 4) educating research teams on key considerations for including older adults in research; and, 5) catalyzing research innovations to increase representation in, and the relevance and translational potential of, research for improved clinical and community-based practice. OARS, whose average age is 69 (range: 58-80), diversify our research workforce and enhance our capacity to include older adults -- an underrepresented population -- in clinical research. This initiative is advancing our Age-Friendly University work to promote encore careers, foster intergenerational learning, and catalyze research innovation. Ultimately, we aspire to increase the generalizability of research findings to improve healthcare and health outcomes for older adults by promoting the inclusion of older adults in our research workforce and in research studies to enhance the benefits to older adults of new drugs, devices and behavioral health interventions. During this session, we will share our training curriculum, how we created pathways to employment and our program outcomes.

